# Layer-specific lipid signatures in the human subventricular zone demonstrated by imaging mass spectrometry

**DOI:** 10.1038/s41598-018-20793-4

**Published:** 2018-02-07

**Authors:** Mandana Hunter, Nicholas J. Demarais, Richard L. M. Faull, Angus C. Grey, Maurice A. Curtis

**Affiliations:** 1 Centre for Brain Research, University of Auckland, Auckland, 1023 New Zealand; 2 Department of Pharmacology and Clinical Pharmacology, Faculty of Medical and Health Sciences, University of Auckland, Auckland, 1023 New Zealand; 3 School of Biological Sciences, Faculty of Science, University of Auckland, Auckland, 1010 New Zealand; 4 Department of Anatomy and Medical Imaging, Faculty of Medical and Health Sciences, University of Auckland, Auckland, 1023 New Zealand; 5 Department of Physiology, Faculty of Medical and Health Sciences, University of Auckland, Auckland, 1023 New Zealand

## Abstract

The subventricular zone is a key site of adult neurogenesis and is also implicated in neurodegenerative diseases and brain cancers. In the subventricular zone, cell proliferation, migration and differentiation of nascent stem cells and neuroblasts are regulated at least in part by lipids. The human subventricular zone is distinctly layered and each layer contains discrete cell types that support the processes of neuroblast migration and neurogenesis. We set out to determine the lipid signatures of each subventricular layer in the adult human brain (n = 4). We utilised matrix-assisted laser desorption/ionisation (MALDI) imaging mass spectrometry and liquid chromatography-mass spectrometry to characterise the lipidome of the subventricular zone, with histology and microscopy used for identifying anatomical landmarks. Our findings showed that the subventricular zone was rich in sphingomyelins and phosphatidylserines but deficient in phosphatidylethanolamines. The ependymal layer had an abundance of phosphatidylinositols, whereas the myelin layer was rich in sulfatides and triglycerides. The hypocellular layer showed enrichment of sphingomyelins. No discrete lipid signature was seen in the astrocytic ribbon. The biochemical functions of these lipid classes are consistent with the localisation we observed within the SVZ. Our study may, therefore, shed new light on the role of lipids in the regulation of adult neurogenesis.

## Introduction

Lipids are highly abundant in the brain, where they show great diversity of structure and function. In the brain, lipids perform a broad range of functions including membrane structure, metabolism, cell proliferation and survival, transcriptional regulation and intracellular signaling. However, their overwhelming structural diversity has relegated lipids to the least understood ‘ome’ of biology, with bioanalytical and computational tools to correct this deficit only now maturing. Lipids found in the brain fall into three major categories, defined by their structure and chemistry: cholesterol, phospholipids and sphingolipids^1^. The phospholipid category includes the following classes of lipids: phosphatidylcholines (PC), phosphatidylethanolamines (PE), phosphatidylinositols (PI), phosphatidylserines (PS). The sphingolipid category contains sphingomyelins (SM), cerebrosides, ceramides (Cer), sulfatides (ST), and gangliosides (GM). In this study, we investigated the lipid signature of the largest area of plasticity in the adult human brain, the subventricular zone (SVZ), in which we have no knowledge of the specific lipids, or indeed the classes of lipids, that are present. The role of lipids in stem cell biology and plasticity is becoming increasingly appreciated^2^. For instance, lysophosphatidic acid, a simple glycerophospholipid, may act as a potent neuromodulator by altering membrane potential, thereby influencing neural progenitor fate^3^. The same lipid also acts as a chemorepulsive molecule to cause neurite retraction and cell rounding, influencing the migration of neural progenitors and nascent neurons during corticogenesis^4,5^. Knobloch *et al*. highlighted the requirement for *de novo* lipogenesis in adult neurogenesis, where the rate-limiting lipogenic enzyme, fatty acid synthase (FASN), shows higher expression in proliferating neural stem cells (NSCs) than their differentiated progeny both *in vitro* and *in vivo*^6^. In the same study, hierarchical clustering of lipid metabolomes successfully discriminated proliferating from non-proliferating NSCs derived from the murine SVZ and dentate gyrus, while the small molecule FASN inhibitor orlistat provided concentration-dependent inhibition of NSC proliferation as assessed by bromodeoxyuridine uptake. Transcriptomic studies identified lipid metabolism genes as among the most differentially expressed between populations of activated and quiescent NSCs. Single-cell RNA sequencing similarly revealed lipid biosynthesis and fatty acid metabolism genes to be highly expressed in NSCs derived from the SVZ^7,8^. Deranged fatty acid homoeostasis in the SVZ of Alzheimer’s disease brains has also been observed, where early lipid accumulation impairs neural progenitor activity^9^. Collectively, such studies suggest that coordination of lipid synthesis and metabolism is involved in regulating the transition of NSCs from a quiescent (non-proliferating) to an active (proliferating) state. In light of these findings, and given that NSCs are highly responsive to external stimuli, we hypothesised the existence of a specialised lipid niche in the SVZ.

Adult neurogenesis occurs in the SVZ of the lateral ventricles and in the dentate gyrus of the hippocampus. This process requires the regulation of an ensemble of cellular programmes including the proliferation of NSCs, lineage commitment of neural progenitors, migration and differentiation into functional neurons and their survival and integration into existing brain circuits. Neurogenesis is modulated by signals arising cell-autonomously and extrinsically from the niche in which NSCs reside, indicating the high responsiveness of NSCs to environmental molecular cues. Neurogenic responses of the SVZ are perturbed under disease conditions and have been studied intensively in the context of ageing, neurodegenerative diseases, psychiatric disorders and brain injury^10,11^. Moreover, a growing body of evidence supports the view that SVZ-resident NSCs can give rise to malignant brain tumours such as glioblastoma multiforme and subependymomas^12–14^. Understanding the cues that are present in the neurogenic niche and how they regulate neurogenesis is, therefore, an important objective. Furthermore, given the evident differences in the human SVZ relative to other animals, studying these phenomena directly in humans is vital^15,16^.

The human SVZ is comprised of four anatomically distinct layers – a superficial ependymal layer (lamina I) that interfaces with the ventricular cavity, a hypocellular layer (lamina II) that contains few cell bodies but houses a dense network of glial fibrillary acid protein (GFAP)-positive astrocytic processes, an astrocytic ribbon with resident NSCs (lamina III) and finally a heavily myelinated zone (lamina IV) that transitions to the underlying parenchyma of the caudate nucleus (CN)^17,18^. Notably, processes of stem-cell-like astrocytes in the third lamina occasionally intercalate with multi-ciliated ependymal cells in the first layer, where they may be involved in the reception of soluble signals from the ventricular cerebrospinal fluid (CSF)^19^. The first lamina, therefore, includes both ependymal cell bodies and astrocytic processes. The SVZ laminae exhibit intimate functional interactions but differ in their cellular compositions and biological properties. To investigate the lipidome of the SVZ in the adult human brain, we utilised matrix-assisted laser desorption/ionisation (MALDI) imaging mass spectrometry (IMS). MALDI IMS provides an ideal approach to the high-throughput analysis of lipid neurochemistry by simultaneously interrogating the relative abundance and spatial distribution of many ionised species. Moreover, the small raster widths achieved by the modern commercial MALDI IMS instruments provide high resolution and thus unique opportunities to investigate the macromolecular composition of the SVZ. Here, we report a comprehensive analysis of the lipidome of the SVZ in the human brain and demonstrate that the most abundant lipid classes in each layer align closely with the specific function of that layer.

## Results

### The human SVZ has a distinct lipidomic signature

**Figure 1:**
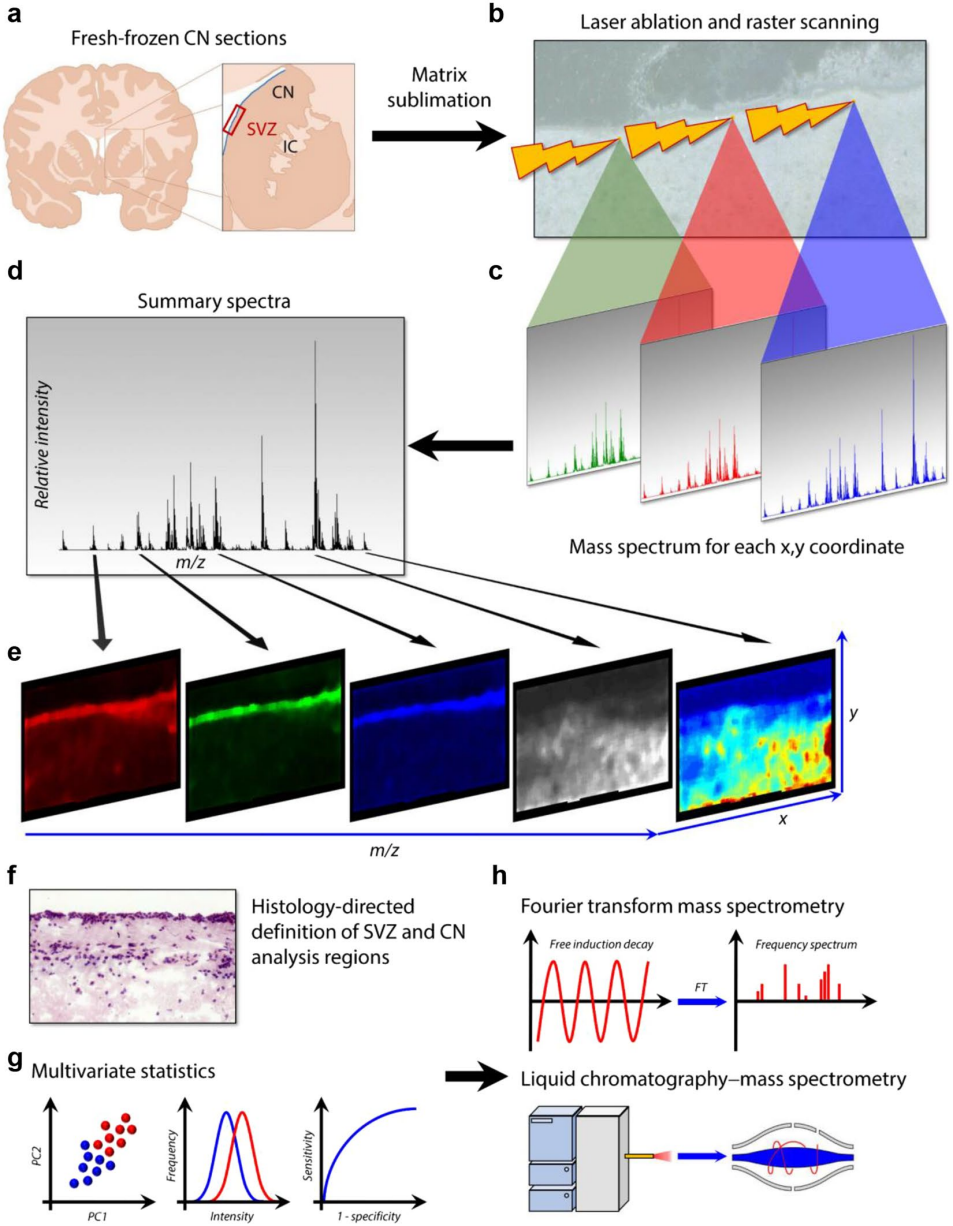
Schematic overview of the experimental approach. (**a**) Matrices were applied to fresh frozen CN sections sampled from the medial aspect of the SVZ indicated by the red rectangle. IC = internal capsule, CN = caudate nucleus. (**b,c**) MALDI imaging was performed with 10 µm raster scanning in x and y directions. (**d,e**) Summary spectra yielded ion intensity maps for hundreds of lipid analytes. (**f**) Post-MALDI stained sections were co-registered with MALDI images to define SVZ and CN analysis regions. (**g**) Multivariate statistics were used to identify ions with differential abundance in the SVZ relative to the CN. (**h**) The corresponding lipids were identified my FT-ICR mass spectrometry and liquid chromatography-tandem mass spectrometry.

**Figure 2:**
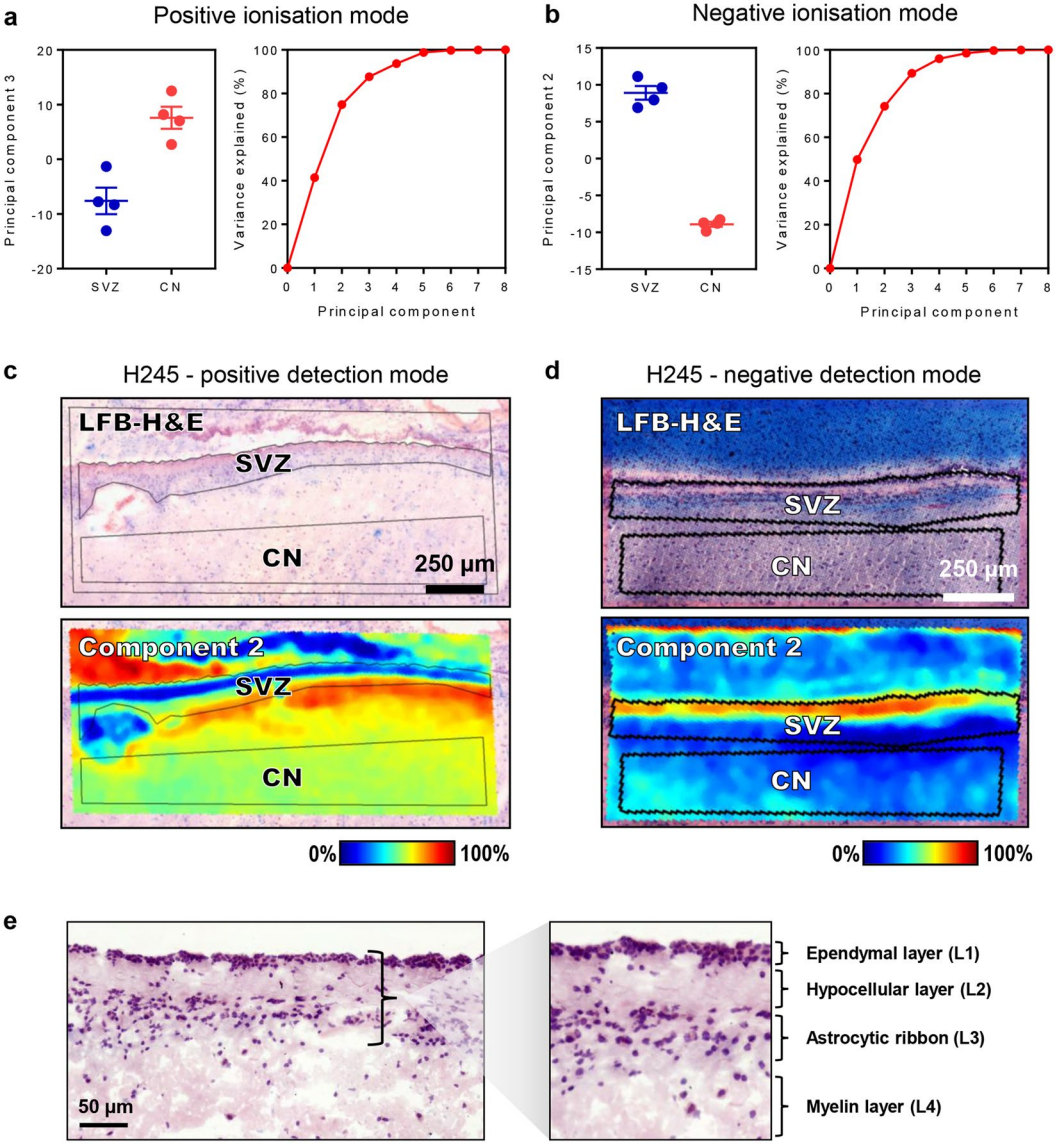
The human SVZ has a distinct lipidomic signature. (**a,b**) Principal component analysis of the lipidomic signature of the SVZ and CN in positive and negative ionisation mode. Principal components were computed from mean intensity data for each m/z signal within histologically-defined analysis regions corresponding to the SVZ and CN for each case. The principal components providing the highest ratio of inter- to intra-group variance in positive and negative polarities are plotted, where groups corresponded to the four SVZ regions and four CN regions. Error bars represent intra-group means ± standard error of the mean. The cumulative fraction of variance in the MALDI-TOF data that is explained by each principal component is shown. (**d,e**) Intensity heat maps of the 2^nd^ principal component in representative sections, showing spatial correlation between the intensity of the component and the anatomy of the SVZ in positive and negative ionisation modes. Principal components for (**c**) and (**d**) were computed using individual spectra within sections as the unit of analysis. Scale bar: 0.5 mm. (**e**) HE-LFB staining illustrating the anatomical structure of the SVZ and its four constituent layers. Scale bar: 50 µm.

To characterise the lipidome of the SVZ, MALDI-TOF (time of flight) imaging of CN sections containing the SVZ from four neurologically-normal cases was performed. IMS was conducted at the highest achievable resolution (i.e. 10 µm raster width with minimum laser diameter) within an imaging region encompassing a medial section of the SVZ and a ventral area of the CN parenchyma. Analysis regions corresponding to the SVZ and CN were defined by reference to histological haematoxylin, eosin and luxol fast blue (HE-LFB) stains and the spectral data for these two regions were compared individually within sections then collectively between cases. Fourier transform-ion cyclotron resonance (FT-ICR) mass spectrometry (MS) and liquid chromatography-tandem mass spectrometry (LC-MS/MS) techniques and a comprehensive statistical pipeline were employed to identify lipids that were consistently either abundant or scarce in the SVZ relative to the parenchyma of the CN (see Fig. 1 for methodology). MALDI-TOF IMS detected 649 and 379 discrete m/z signals in positive and negative ionisation modes, respectively. In each ionisation mode, the principal component analysis identified eight components that explained 100% of the variance between the SVZ and CN regions (Fig. 2a,b) based on their respective summary spectra, which are mean spectra generated from the addition of all mass spectra in each anatomically-defined region. Notably, the SVZ and the CN had marked and consistent differences in the intensity of prominent components, indicating distinct lipid profiles and confirming differences in lipid ion abundances between these two regions to be a major source of variance in the MALDI IMS data. This observation was extended by performing a second series of within-sample principal component analyses (one for each case) to identify sources of spectral variance between individual laser ablation points. Twenty components were obtained for data collected in positive and negative ionisation modes; in the latter case, 20 components explained near-complete (>99.995%) variance in the data, whereas the former approached an asymptote at 70% of variance explained (Fig. S1). Overlaying the resulting component intensity maps with HE-LFB stains (Fig. 2c,d, Fig. S1a,b) identified anatomic co-localisation of multiple components with the SVZ, further confirming lipidomic specialisation of this region to be a major feature of the MALDI IMS data. Interestingly, the greatest difference in component profiles was evident between the ependymal and myelin layers (e.g. Fig. 2d, Fig. S1b; see Fig. 2e for the anatomical structure of the SVZ laminae), suggesting that these two laminae are most distinct in their lipid profiles, whereas the hypocellular and astrocytic layers were less clearly discriminated by principal component analysis.

### Identification of SVZ-associated lipids

**Figure 3:**
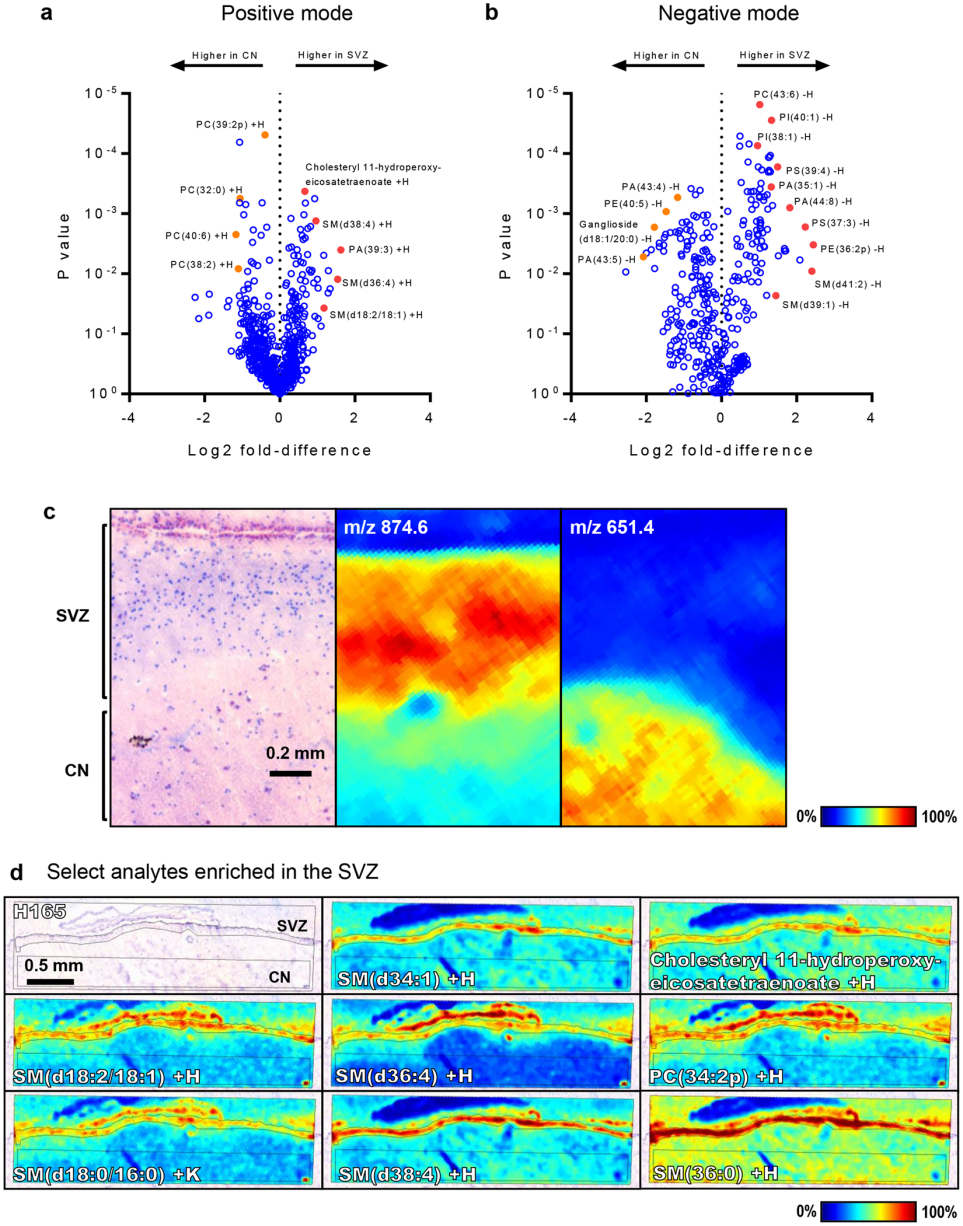
Lipid ions with differential abundance in the SVZ and the CN. (**a,b**) Volcano plots of individual m/z signals in positive and negative polarity, with log2-transformed fold differences (SVZ-CN ratio of mean intensities; mean of four cases) plotted against P-values arising from one-sample t-tests. Lipid species that are discussed in the text are arbitrarily marked in red here and the assignments for these are indicated. (**c**) Anatomy of the SVZ and CN in a representative case, with examples of m/z signals found to co-localise with each region. Scale bar: 0.2 mm. (**d**) Ion intensity maps are shown for select analytes with high local abundance in the SVZ as compared to the CN in a representative case. Scale bar: 0.5 mm.

To elucidate the specific molecules that contributed to the SVZ lipid profile, a pipeline for the statistical analysis of the MALDI IMS data was developed by integrating three complementary approaches: 1, co-localisation analysis (Pearson correlation for m/z signals anatomically associated with the SVZ); 2, receiver operating characteristics (ROC; favouring m/z signals providing maximal specificity and sensitivity to discriminate the SVZ from the CN across all intensity values of the analyte); 3, one-sample t-tests with Benjamini-Hochberg correction for multiple hypotheses (favouring m/z signals with differing mean intensities between SVZ and CN summary spectra). The statistical metrics from each of these approaches (correlation coefficients, areas under the ROC curves (AUC) and FDR-corrected *P*-values, respectively) were collated to derive a list of m/z signals either consistently abundant or scarce in the SVZ relative to the CN. The identities of the corresponding lipids were then assigned by accurate mass matching and structural analysis generated by on-tissue MALDI-FT-ICR and LC-MS/MS analysis of tissue extracts, respectively. Isotopic species were excluded by comparing spatial distributions and predicted vs. observed intensities relative to monoisotopic peaks. Initial analysis of each SVZ and CN as discrete units (i.e. without discriminating the constituent laminae of the SVZ) identified 46 lipids with consistently higher abundance in the SVZ relative to the CN across both detection modes (Fig. 3, Fig. S2–S3, Tables S1–S2), whereas 42 lipids showed higher abundance in the CN (Tables S3–S4). Several m/z signals had statistically significant differences between the SVZ and CN but their identities could not be robustly assigned and were therefore excluded from results tables. To visualise the magnitude and statistical significance of lipid abundance/scarcity in the SVZ relative to the CN, log2 fold-changes in mean intensity between these two regions were plotted against *P*-values from one-sample t-tests to derive volcano plots (Fig. 3a,b). The SVZ was notably enriched in several species of SMs and PS’s, with up to 3-fold higher abundance detected for examples from each lipid class and a number of such species providing near-perfect discrimination between SVZ and CN (areas under the ROC curve approaching 1; Tables S1-S2). Notably, some species were specific to the ependymal layer of the SVZ (e.g. SM(d31:2) −H; Fig. S3), whereas others co-localised with the myelin layer (e.g. TG(58:14) −H; Fig. S3) or were uniformly abundant throughout the SVZ (e.g. PS(36:1) −H; Fig. S3). While many lipids were specific to the SVZ, others were also abundant in regions of the septum (e.g. PC(34:2p) +H; Fig. S2), which was incidentally imaged due to its proximity to the SVZ within the tissue blocks. Several lipids were common to the ependymal layer of the SVZ and the parenchyma of the CN but absent in the hypocellular, astrocytic and myelin layers (e.g. PI(38:4) −H; Fig. S3). The CN itself was replete in PCs, with 12 such lipids showing robust associations, highly-significant discriminatory power and 2- to 5-fold higher abundance relative to the SVZ (Fig. 3a–b, Tables S3–S4). Interestingly, two isomers of the ganglioside GM1 (m/z 1544.8 and 1573.0) and two neutral glycosphingolipids were also scarce in the SVZ, with higher abundance in the CN (Table S4, Fig. S3).

### Each SVZ layer has a unique lipidomic signature

**Figure 4:**
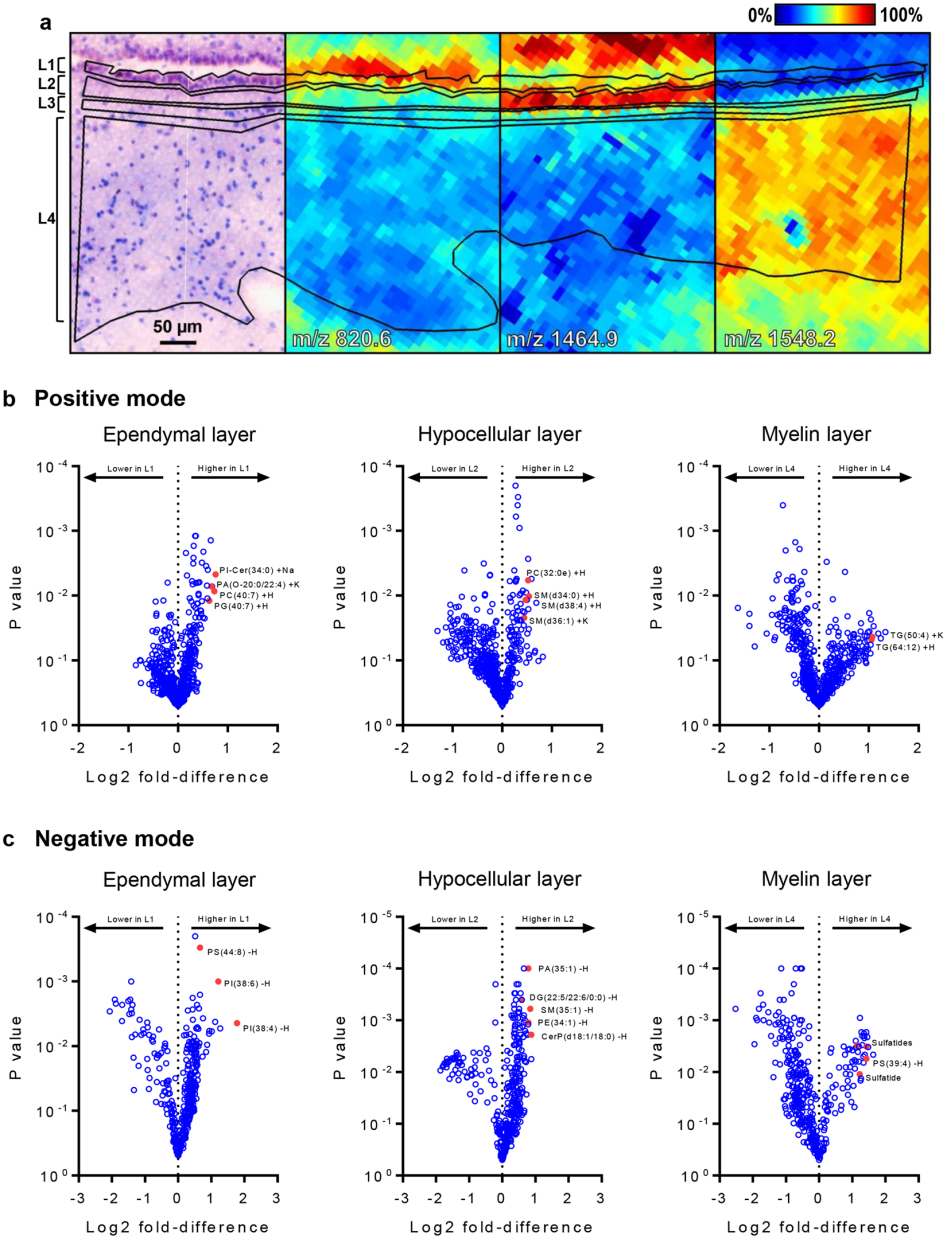
The lipidomic architecture of the constituent layers of the SVZ. (**a**) Histology-guided delineation of the four SVZ layers as analysis regions in SCiLS Lab software, with example m/z signals shown that were found to co-localise with the ependymal layer, hypocellular layer or the myelin layer. Scale bar: 50 µm. (**b,c**) Volcano plots of individual m/z signals in positive and negative polarity with log2 transformed fold differences plotted against P-values arising from one-sample t-tests. Summary spectra for each layer (including the astrocytic ribbon) were obtained and log2 fold-differences and t-test P-values derived by comparing, for each m/z signal, its intensity value in the summary spectrum for each layer with the mean intensity of the summary spectra corresponding to the three other SVZ layers. L1 = ependymal layer; L3 = hypocellular layer; L4 = myelin layer. Lipid species that are discussed in the text are arbitrarily marked in red here and the assignments for these are indicated.

**Figure 5:**
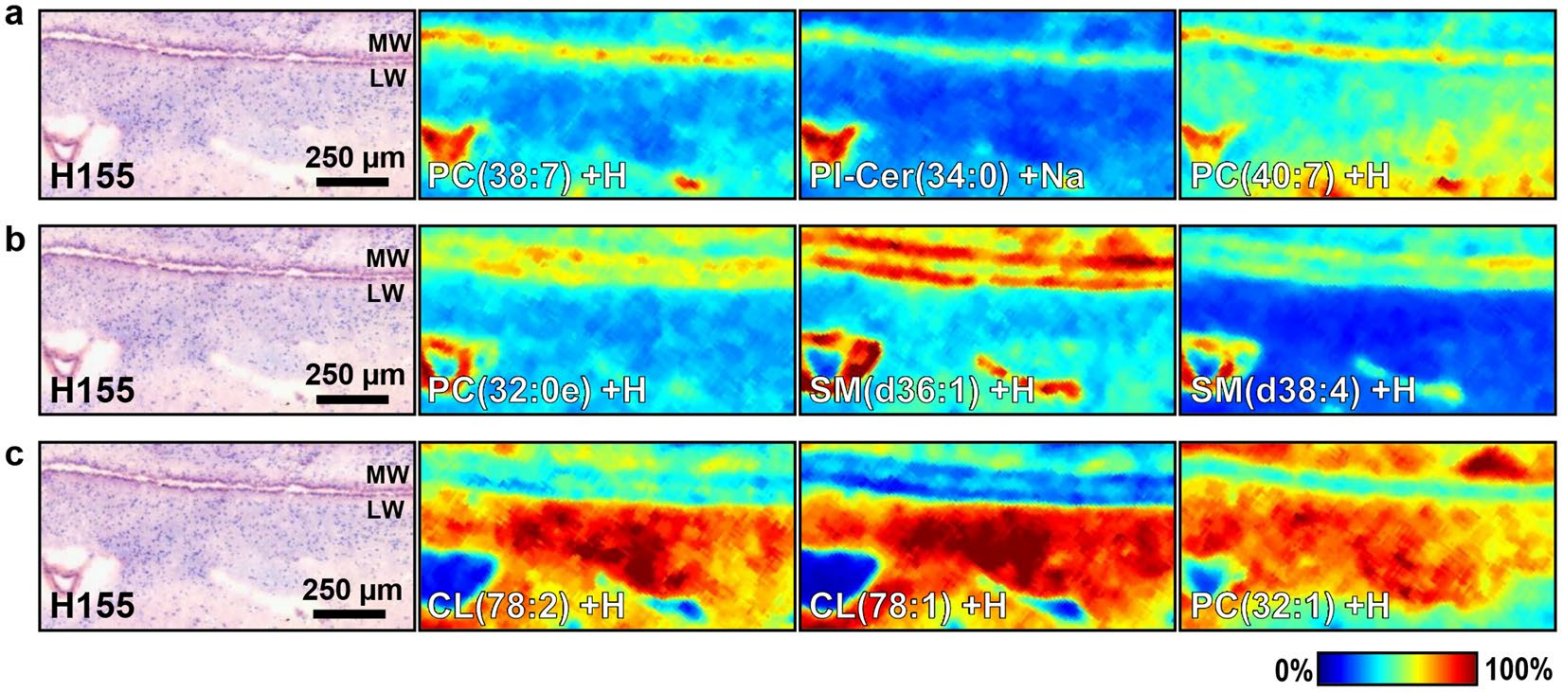
Ion intensity maps of select analytes associated with SVZ layers. Spatial distributions are shown for select lipid ions that were identified as statistically co-localised with the ependymal layer (**a**), the hypocellular layer (**b**) and the myelin layer (**c**), respectively, when compared to the three other SVZ layers in each circumstance. Lipid signal intensities in each MALDI sampling position are scaled to the maximum signal intensity for each lipid across all sampling positions, with mild denoising applied to the images within the SCiLS Lab software. The positions of the ependyma of the lateral wall (LW) and the medial wall (MW) of the ventricle are indicated within post-MALDI HE-LFB stains. Scale bar: 250 µm. PC = phosphatidylcholine, CL = cardiolipin, SM = sphingomyelin, PI-Cer = ceramide phosphoinositol.

Next, the lipid composition in each of the four SVZ layers was studied. HE-LFB images were used as a reference and co-registered to the optical scans of prepared MALDI imaging sections that were utilised in the MALDI imaging data collection phase (Fig. 4a). The lipidomic composition of each layer was then compared to the remaining three using co-localisation analysis and t-statistics. Since the surface area differed for each of the laminae, t-tests were performed by first deriving summary spectra for each layer and then comparing peak intensities from the summary spectrum of the layer under consideration to the mean peak intensities from summary spectra of the remaining three layers. Using summary spectra in this manner, each layer was afforded equal weighting in the statistical analysis. Combined assessment of the co-localisation coefficients and t-statistics separated groups of lipids that were robustly associated with the ependymal, hypocellular or myelin layers (Fig. 4b,c and Fig. 5). No distinct lipid profile was observed for the astrocytic ribbon, potentially reflecting difficulties in defining the boundaries of this region within some of the tissue sections. The ependymal layer was enriched for 22 lipids that could be confidently assigned (Table S5, Fig. 4b,c and Fig. 5a). The most striking observation was a markedly higher abundance of PIs, with ten such lipids with varying acyl chain configurations showing strong ependymal associations. As the ependyma of the ventricular medial wall was inadvertently imaged in 2 of 4 cases, we undertook a preliminary assessment of whether the lipids associated with the ependyma of the SVZ were also present in the ependyma of the medial wall (i.e. multi-ciliated ependymal cells outside the neurogenic niche. This analysis suggested the identified lipid profile to be specific to the ependyma of the SVZ (Fig. S4), though this requires further investigation. The hypocellular layer was enriched for 22 different lipids spanning a range of structural classes (Table S6, Fig. 4b,c and Fig. 5b), notably including PEs, at least eight examples of which were abundant in this region. The myelin layer was enriched for 16 lipids (Table S7, Fig. 4b,c and Fig. 5c). Notable of these and unique among the SVZ layer were high abundances of five species of STs, four triglycerides (TGs) and one saturated cardiolipin.

## Discussion

This study provides comprehensive analysis and characterisation of the lipid architecture of the human SVZ, revealing a lipid profile that can discriminate the SVZ from the neighbouring CN and high degrees of specialisation within each constituent SVZ layer. Two complementary analysis approaches were used in this study. Firstly, the SVZ was compared to the adjacent parenchyma of the CN to identify lipids enriched within the SVZ in its gross anatomy. Secondly, we characterised the lipidomic substructure within the SVZ, identifying lipids co-localised with each layer. This dual approach enabled us to identify associations that would have been impervious to one method alone.

The SVZ- and layer-specific distributions of lipids observed in our study aligned with the known function of each of the SVZ layers. For instance, SMs were identified as highly abundant in the SVZ relative to the CN (Fig. 3, Tables S1 and S2). SMs are prevalent sphingolipids enriched in the plasma membrane, where they play key roles in transmembrane signaling. Indeed, SM and the products of its iterative hydrolysis – ceramide and sphingosine (and the 1-phosphate derivative thereof) – are central mediators of sphingolipid signalling, which regulates diverse cellular processes including mitogenic signal transduction, self-renewal, neural differentiation, apoptosis, cell cycle kinetics, migration of SVZ-born postnatal neuroblasts to the olfactory bulb and the genesis of primary and motile cilia^20–25^. Biosynthetic regulation of the SM-ceramide-sphingosine axis, therefore, provides a rheostat for many cell biological processes that are central to the function of the SVZ. Conversely, less requirement for processes such as cell survival, proliferation, migration and differentiation may be expected in the CN.

In the CN, we observed a higher abundance of several more complex glycosphingolipids, including gangliosides – a lipid class that is characterised by the presence of sialic acid residues and is typically embedded within the plasma membrane. Due to their sialoglycan components, which constitute 75% of the total conjugated sialic acid in the brain, gangliosides actively mediate plasticity via cell-cell recognition, cell adhesion, motility and growth^26–28^. In the mature brain, gangliosides are principally concentrated in the grey matter. Their observed presence in the CN is, therefore, unsurprising. However, it is of interest to note that despite their well-recognised role in regeneration, plasticity and neurodevelopment^29^, the particular ganglioside species observed here were less abundant in the SVZ than the CN. We acknowledge that the observed isoforms of GM1 (d18:1/C18:0) and (d20:01/C18:0) may arise from loss of sialic acid from ganglioside GD1 as result of in-source fragmentation. Nevertheless, both GM1 and GD1 perturbations have been observed in neurodegenerative disorders such as Huntington’s^30^, Parkinson’s^31^ and Alzheimer’s^32^ diseases. Indeed, our laboratory has previously observed GM1 to be lower in the dentate gyrus – the other neurogenic niche in adults – of postmortem brains from Alzheimer’s disease patients^33^. It would be of interest to investigate the abundance of gangliosides in the SVZ under neurodegenerative conditions using MALDI IMS.

The SVZ is superficially comprised of a monolayer of ependymal cells that interface with ventricular CSF. As such, the ependyma of the SVZ may play an important role in regulating neurogenesis and maintenance of SVZ homoeostasis in response to signalling molecules circulating in the CSF. Ependymal cells also possess motile cilia, which include the central pair of microtubules (9 + 2). The beating of ependymal motile cilia generates a concentration gradient and a directional flow of CSF chemotactic molecules that are essential for the migration of nascent neuroblasts to the olfactory bulb^34^. Ependymal cells have a striking planar polarity that is central in establishing the orientation of ciliary beating and CSF propulsion^35,36^. The primary cilia of radial glia establish planar polarity prior to the transformation of these neural stem cells into their ependymal cell progeny; a process that is then refined by motile cilia in the mature ependymal cells^37^. The ependymal layer was enriched in PIs (Fig. 4, Table S5), which have a role in primary ciliogenesis and establishing planar polarity^37–39^. PIs are also relevant in the formation of motile cilia, as phosphatase-mediated PI(4,5)P_2_ depletion prohibits the formation of mature flagella^40^. It may be that the observed localisation of PIs in the ependymal layer reflects the requirement for genesis and maintenance of motile cilia in these cells, potentially in addition to canonical roles in signaling, membrane organisation and trafficking such as endo- and exocytosis, both of which may be involved in the reception of signals from the CSF. Interestingly, defects in both primary and motile ependymal cilia and signal transduction are seen in hydrocephalus, a common condition associated with excessive CSF accumulation^41^. It would be of interest to investigate whether PIs are perturbed in the SVZ of hydrocephalic brains.

The hypocellular layer, or lamina II, is low in myelin and contains sparse cell bodies – mainly astrocytes – with a network of GFAP-positive processes that contain intermediate filaments and gap junctions^42^. This layer, unique to human SVZ anatomy and absent in rodents, creates a gap between the ependymal layer and the third lamina, the astrocytic ribbon. It remains unclear what function this hypocellular gap serves in human SVZ. Given the prominent astrocytic and ependymal interconnections seen in this lamina, it may serve as a regulatory platform for neuronal function, metabolic homoeostasis, NSC proliferation and differentiation or as a roadway for cellular migration. Indeed, TuJ1-positive (immature neuronal) soma have been reported in the hypocellular layer^42^. We found this lamina to be enriched with SMs (Fig. 4). As discussed, SMs are involved in many cellular processes that would be consistent with a role for the hypocellular layer as an NSC regulatory buffer. We also observed a high abundance of PEs in this layer (Fig. 4). PEs are a principal membrane phospholipid and are major reservoirs of n-3 and n-6 polyunsaturated fatty acids (PUFA). These naturally-occurring PUFA are involved in gap junction coupling, through which astrocytes supply energy to neurons, buffer extracellular K^+^ and glutamate and propagate calcium waves, thereby contributing to the prevention of neuronal insult and to brain homoeostasis^43^. We speculate that the high levels of PEs localised in this layer are likely due to PUFA-dependant gap junction coupling and the supportive function of astrocytes in the SVZ niche.

The third layer – lamina III – is a ribbon of astrocyte cell bodies. A subpopulation of astrocytes in this layer is believed to possess proliferative capacity, generating multipotent progenitors that migrate to their final destination prior to differentiating into neuronal or glial cells. Notably, we were unable to identify lipids specific to the astrocytic ribbon, which may reflect the difficulty of defining its boundaries based solely on histological imaging. However, this may also result from the high heterogeneity of the layer. NSCs represent a hierarchy of cells with nonidentical phenotypes and molecular signatures. The astrocytic ribbon is comprised of a mixture of quiescent and activated NSCs, amplifying progenitors and neuroblasts as well as three discrete populations of astrocytes. Oligodendrocytes are also present, in addition to displaced ependymal cells^17^. This complex cellular milieu may demand less lipidomic specialisation (or rather, a greater diversity of lipids) than seen in other layers.

The myelin layer transits the SVZ to the parenchyma of the ventral CN. Myelin is a highly-specialised and multilamellar material synthesised by oligodendrocytes in the CNS. Myelin encapsulates axons where, due to its low capacitance, it expedites the conduction of neuronal impulses in a saltatory manner. Central to the fulfilment of its function is its high lipid content of over 70%. We identified the myelin layer to be enriched in five species of STs or sulfated galactocerebrosides (GalC; Fig. 4, Table S7), a glycosphingolipid class that is abundant in several organs including the brain^44,45^. Our observation is consistent with previous reports that almost 25% of myelin lipids are comprised of STs and non-sulfated GalC^45^. STs and GalC in myelin typically carry 24:0 or 24:1 acyl chains^46^; notably, these fatty acids were present in four of the five STs we identified (Table S7). GalC is synthesised via the transfer of galactose to ceramide by UDP glycosyltransferase 8 (UGT8)^47^. The importance of GalC was shown in *ugt8* knockout mice, which demonstrate a breakdown of axon insulation and loss of saltatory conduction with the emergence of tremor, splayed hindlimbs and uncoordinated gait^48^. While UGT8 can glycosylate ceramide containing hydroxylated or non-hydroxylated acyl units, it has a higher affinity for the hydroxylated substrate^49^. Accordingly, 2-hydroxylated glycolipids are amongst the most abundant components of myelin^48^ and three of the STs we identified were 2-hydroxylated (Table S7). Mice lacking fatty acid 2-hydroxylase initially synthesise structurally-normal myelin, yet show progressive demyelination and axonal degeneration, indicating the 2-hydroxylated form to be important in long-term maintenance and stabilisation of myelin^50^. STs and GalC are also considered vital to the development of oligodendrocytes, which are key constituents of the myelin layer. Indeed, GalC is one of the earliest markers distinguishing immature oligodendrocytes from their O2A progenitors, while exposure of immature oligodendrocytes to anti-GalC antibodies impedes their maturation^51^.

Four species of TGs were also observed – three polyunsaturated and one monounsaturated – that were co-localised with the myelin layer (Table S7). TGs principally serve as a repository for fatty acyl units that may be liberated by lipases expressed in CNS tissue^52^. TGs themselves and the fatty acids they sequester – particularly PUFA – have been implicated in myelination and NSC biology. Oligodendrocytes isolated from murine^53^, bovine^53^ and porcine brains^54^ synthesise TG in long-term culture, presumably to produce myelin precursors. Adult NSC activity appears to be intimately associated with fatty acid metabolism. Unlike mature neurons and astrocytes, SVZ-resident NSCs do not require glucose to sustain respiration and instead drive the citric acid cycle by beta-oxidation of fatty acids to acetyl-CoA^55^. Accordingly, pharmacological inhibition of beta-oxidation suppresses oxygen consumption and proliferation in NSCs^55^. In isolates from the rat cerebral cortex, proliferating cells primarily oxidise exogenous fatty acids, whereas post-mitotic cultures esterify these units to produce TG^56^. Omega-3 PUFA, which must be obtained from dietary sources, appear to be especially important in NSC regulation. It is noteworthy that the two SVZ-localised TG for which acyl chains could be identified by LC-MS/MS in our study – TG(18:4/18:4/20:5) and TG(18:4/18:4/18:4) – are sources of the omega-3 PUFA eicosapentaenoic acid (EPA) and stearidonic acid (SDA).

In conclusion, the lipid neurochemistry we describe contributes to improving our understanding of adult neurogenesis in the human SVZ and highlights the relevance of lipids in the neurogenic niche, which has been under-appreciated. Although neurogenesis persists throughout life, its capacity declines as a function of age^57,58^. In this regard, it is noteworthy that three of the four cases in our study were more than 60 years old at the time of death. Post-mortem brains from young adults are scarce, though it would be of interest to investigate lipid profiles in a younger human cohort. It will also be of interest to investigate whether the lipidomic signatures we identified are preserved or altered in neurodegenerative diseases that influence the SVZ. Finally, the analytical approaches reported here are readily repurposable to address this and other questions in the burgeoning field of lipidomics.

## Methods

### Tissue collection

Fresh frozen, postmortem human brain tissue from CN blocks of neurologically normal donors was obtained from the Neurological Foundation Douglas Human Brain Bank (Centre for Brain Research, University of Auckland, New Zealand). The tissue used in this study was processed according to the published protocol of Waldvogel *et al*.^59^. In brief, the brain was dissected into blocks, snap-frozen on dry ice and stored at −80 °C. The protocols were approved by the University of Auckland Human Participants Ethics Committee (ref.011654). All tissues were obtained with the informed consent of the families. The presence of intact SVZ on the CN sections was confirmed by performing HE-LFB staining on serial sections immediately adjacent to those used for MALDI imaging. The age, gender, cause of death and postmortem delay of the cases used in this study are provided in Table 1.

**Table 1:** Details of the study cohort. PMD = postmortem delay. SD = standard deviation.

Case	Age (y)	PMD (h)	Sex	Cause of death
H123	78	7.5	M	Ruptured aortic aneurysm
H155	61	7	M	Asphyxia
H165	43	26	F	Nitrogen poisoning
H245	63	20	M	Asphyxia
*Mean*	61	15	—	—
*SD*	14	9	—	—

### Tissue preparation

The tissue blocks were warmed to −20 °C, mounted onto a chuck using Optimal Cutting Temperature (OCT; Sakura Finetek, Torrance, CA), sectioned at 12 µm thickness on a Bright OTF5000 Cryostat (A-M Systems, USA) at −20°C and thaw-mounted onto pre-cooled indium tin oxide-coated MALDI glass slides (Hudson Surface Technology, USA). All sections were obtained from the central region of the rostral-caudal axis of the SVZ. The sections were dehydrated in a vacuum desiccator (Jencons, USA) for 1 hour and then rinsed with ammonium formate (50 mM) as described^60^ to reduce sodium- and potassium-adducted species and to increase signal intensity and the signal-to-noise ratio. For MALDI imaging in positive mode, 2,5-dihydroxybenzoic acid (DHB, Sigma-Aldrich, St Louis, MO) was deposited onto the samples using an in-house vacuum sublimation apparatus for 10 min at approximately 50 mTorr and 110 °C. For MALDI imaging in negative mode, 1,5-diaminonaphthalene (DAN, Sigma-Aldrich, St Louis, MO) was similarly deposited for 5 min at approximately 50 mTorr and 140 °C.

### MALDI-TOF imaging

MALDI imaging experiments were performed using a Bruker ultrafleXtreme (Bruker Daltonics, Bremen, Germany) equipped with a 355 nm Smartbeam II laser operated at 2 kHz, in reflector mode, at an accelerating voltage of +20 kV or −20 kV. External calibration was performed with a series of red phosphorus clusters (1 mg.mL^−1^ in acetonitrile) prior to image acquisition. The laser was set to the minimum beam size. Images were acquired with 150 laser shots/spectrum for negative ion mode and 200 shots/spectrum for positive ion mode, with a raster step size of 10 µm. Data were collected in the m/z range of 320–2000. After imaging, the matrix was removed from the sections using 70% ethanol and hydrated through graded ethanol to distilled, deionised H_2_O before being stained with HE-LFB using standard protocols.

### Alignment and analysis of TOF data

MALDI-TOF spectra were realigned using the Batch Processing macro of flexAnalysis v3.3 (Bruker Daltonics, Bremen, Germany) with a custom algorithm. Briefly, peak picking was performed on unaligned spectra and a matrix of the detected m/z values, censored to an accuracy of 0.1 unit, was read into the *R* statistical environment, where the table function was used to identify the most frequently occurring values. The latter were used to define a mass control list spanning the full m/z range. Deviations from control masses in actual m/z values for all spectra were measured and assignment tolerances in flexAnalysis set accordingly. Calibration then proceeded using the ‘internal calibration’ routine within flexAnalysis. Calibrated spectra were read into SCiLS Lab 2016b (Bruker Daltonics, Bremen, Germany), where baseline subtraction was achieved using the convolution algorithm and data were normalised using the total ion count. Peak finding was performed for each 9^th^ spectrum (to manage processing times) with interval widths of ±0.12 Da (positive mode) or 0.15 Da (negative mode) and a maximum of 300 signals per spectrum. Post-MALDI HE-LFB images were co-registered with the IMS images and used to define the SVZ and CN as analysis regions. The abundances of lipid ions within regions were compared using spatial co-localisation (Pearson correlation), areas under receiver operating characteristic (ROC) curves and one-sample t-tests. For the latter, the signal-wise SVZ-CN ratio of mean ion intensities from the summary spectra for each region was computed. The resulting quotients were log2 transformed and t-tests with Benjamini-Hochberg correction for multiple hypotheses were performed to assess deviation of the statistic from 0. For each analytical approach, intra-case SVZ-CN comparisons were made and the inter-case mean ± standard error of the mean of each statistical metric is reported.

### Fourier transform ion cyclotron resonance (FT-ICR) imaging mass spectrometry (IMS)

Sections from one case were prepared and DHB or DAN matrices applied as described above. FT-ICR IMS of the SVZ was performed using a Bruker 7 T solarix-XR mass spectrometer (Bruker Daltonics, Bremen, Germany) with MALDI ionisation and at 20 µm spatial resolution. Spectra were collected in the m/z range of 150–2000. FT-ICR data were read into flexImaging v4.1 (Bruker Daltonics, Bremen, Germany) and normalised using the root mean square method. Peak centroids were taken as accurate masses to facilitate the assignment of lipid identities.

### Liquid chromatography-tandem mass spectrometry (LC-MS/MS)

Methods used for lipid extraction and LC-MS/MS analysis of extracts from caudate nucleus sections from three human subjects are detailed in the Supplementary Materials.

### Lipid assignments

Lipid assignments were made by integrated assessment of accurate masses obtained by FT-ICR and fragment ion spectra from LC-MS/MS. FT-ICR peaks corresponding to TOF m/z signals from SCiLS Lab were identified by comparing both m/z values and spatial distributions. Where FT-ICR peaks corresponded to LC-MS/MS analytes within an error limit of 10 ppm, the assignment made based on LC-MS/MS spectra was accepted. In the absence of an LC-MS/MS signal, assignments were made by database searching of accurate masses using LIPID MAPS^61^. The closest assignment within an error limit of 10 ppm was accepted, with manual exclusion of species of plant, fungal or prokaryotic origin. Where ≥2 assignments could not be discriminated, all possibilities are reported. The existence of isotopic peaks, where ≥2 signals were separated by intervals of 1 mass unit, was investigated by comparing spatial distributions and relative signal intensities. Where distributions were uniform and the relative intensities of first and second ^13^C isotope peaks matched predictions arising from the assignment of the monoisotopic peak, the former were excluded from subsequent analysis.

## Electronic supplementary material

Dataset 1
