# Supplementary material for: Layer-specific lipid signatures in the human subventricular zone demonstrated by imaging mass spectrometry
Source: Sci Rep. 2018 Feb 7;8:2551. doi: 10.1038/s41598-018-20793-4 (PMC5803191; doi:10.1038/s41598-018-20793-4)
Supplement: Supplementary file 1 — Dataset 1 [file 41598_2018_20793_MOESM1_ESM.pdf]

Supplementary Materials for

Layer-specific lipid signatures in the human subventricular zone demonstrated by imaging mass spectrometry

Mandana Hunter, Nicholas J. Demarais, Richard L.M. Faull, Angus C. Grey and Maurice A. Curtis*

*Corresponding author email: m.curtis@auckland.ac.nz

Supplementary Methods

Liquid chromatography–tandem mass spectrometry (LC–MS/MS). Tissue was sectioned from four cases for analysis of lipid content by LC–MS/MS, transferred cold into sterile glass vials, weighed and flash frozen in liquid nitrogen. Lipid extraction was performed according to a modified method of Bligh and Dyer[1] with the use of dichloromethane (Sigma-Aldrich, St Louis, MO). Extraction of lipids was effected by vortexing for 20 s in 190 μL methanol, after which 320 μL dichloromethane was added and samples vortexed again for 20 s prior to the addition of 120 μL of water. The samples were vortexed again for 10 s then allowed to equilibrate for 10 min at room temperature before centrifugation at 8000 g for 10 min at 10°C. The lower, lipid-rich dichloromethane fraction was then isolated and the solvent evaporated to dryness under vacuum. Samples were reconstituted in 300 μL acetonitrile/2-propanol/H_2O (65:30:5 v/v/v). The samples were analysed by ultra-high pressure liquid chromatography–MS/MS using an Accela 1250 Pump coupled to a Q Exactive Orbitrap mass spectrometer (Thermo Fisher Scientific, Waltham, MA) operating in heated electrospray ionisation mode. The column and samples were maintained at temperatures of 45°C and 4°C, respectively. Injection volume was 5 μL. Chromatographic separations were performed using an Accucore C18 150 x 2.1 mm, 1.6 μm solid core column (Thermo Fisher Scientific, Waltham, MA). Mobile phases were acetonitrile/H_2O (60:40, v/v) + 0.1% formic acid + 10 mM ammonium formate (Solvent C) and isopropanol/acetonitrile (90:10, v/v) + 0.1% formic acid + 10 mM ammonium formate (Solvent D). Reversed-phase LC was conducted and a flow rate of 0.26 $mL.min^{-1}$ was applied with a gradient elution of 32% to 97% solvent D over 21 min. Centroid MS scans were acquired in the mass range of 133.4-2000 m/z using the Orbitrap mass spectrometer with mass resolution ΔM of 70,000 (full width at half maximum, FWHM, as defined at m/z 200), automatic gain control of 3e6, injection time of 100 ms, sheath gas flow rate of 25 units, auxiliary gas flow rate of 15 units, sweep gas flow rate of 0 units, capillary temperature of 250°C, S-lens radiofrequency 50% and heater temperature 350°C. Spray voltage was 3.5 kV for negative ions and 3.1 kV for positive ions. Parallel reaction monitoring acquisition was performed with a mass resolution of 17.5, automatic gain control of 2e4, injection time of 100 ms, isolation window of 2.0 m/z and normalised collision energy of 30%, 60% and 90% with combined MS/MS spectra resulting. Parent ion assignments were made using LipidSearch software (Thermo Fisher Scientific, Waltham, MA).

1. Bligh, E. G. & Dyer, W. J. A rapid method of total lipid extraction and purification. *Can. J. Biochem. Physiol.* **37,** 911–917 (1959).

Supplementary Figures

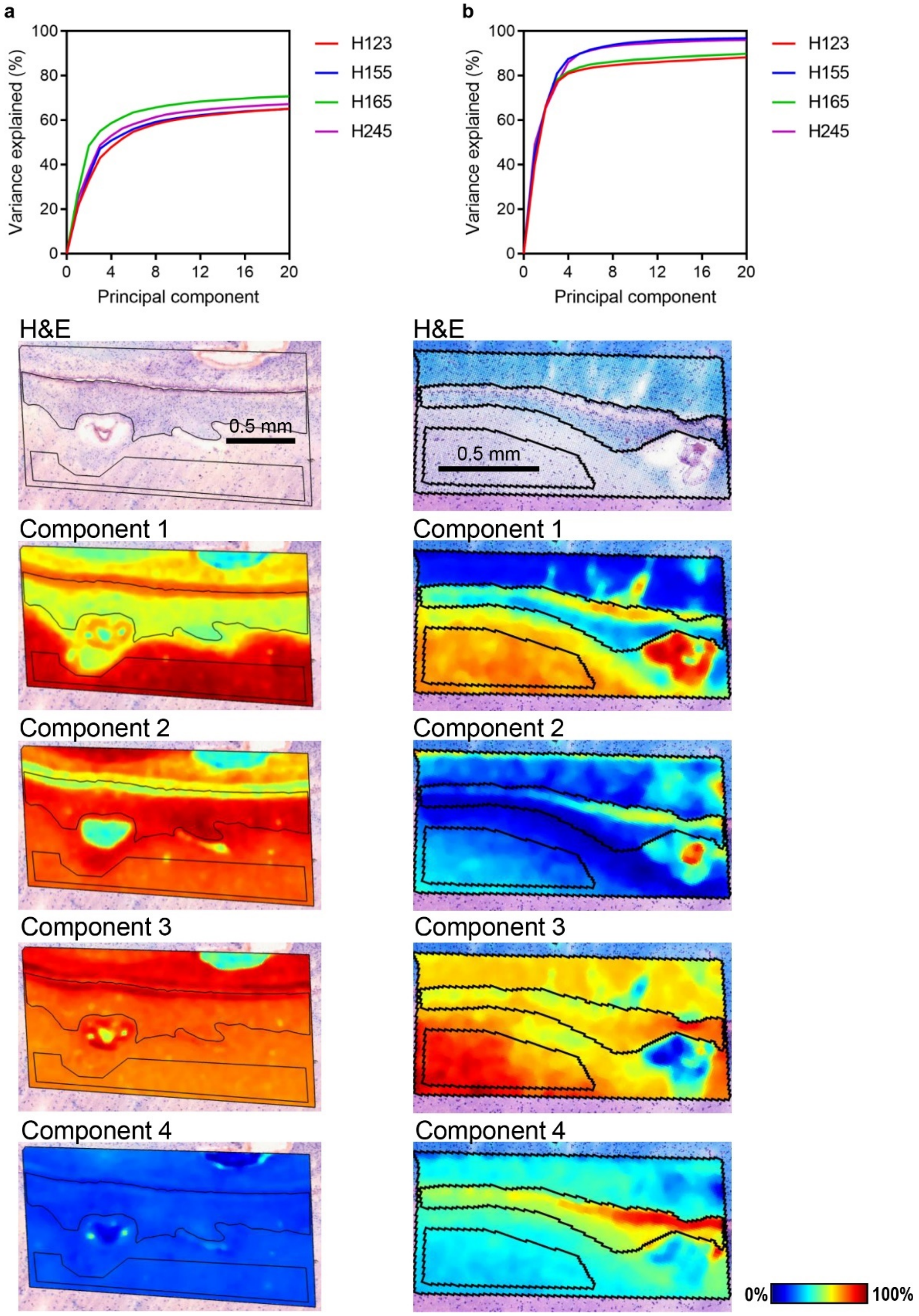

Figure S1. Principal component analysis of mass spectra within individual sections. Cumulative variance explained by principal components (PCs) and heat maps of the first four PCs in a representative case (H155) in positive (a) and negative (b) ionisation modes are shown.

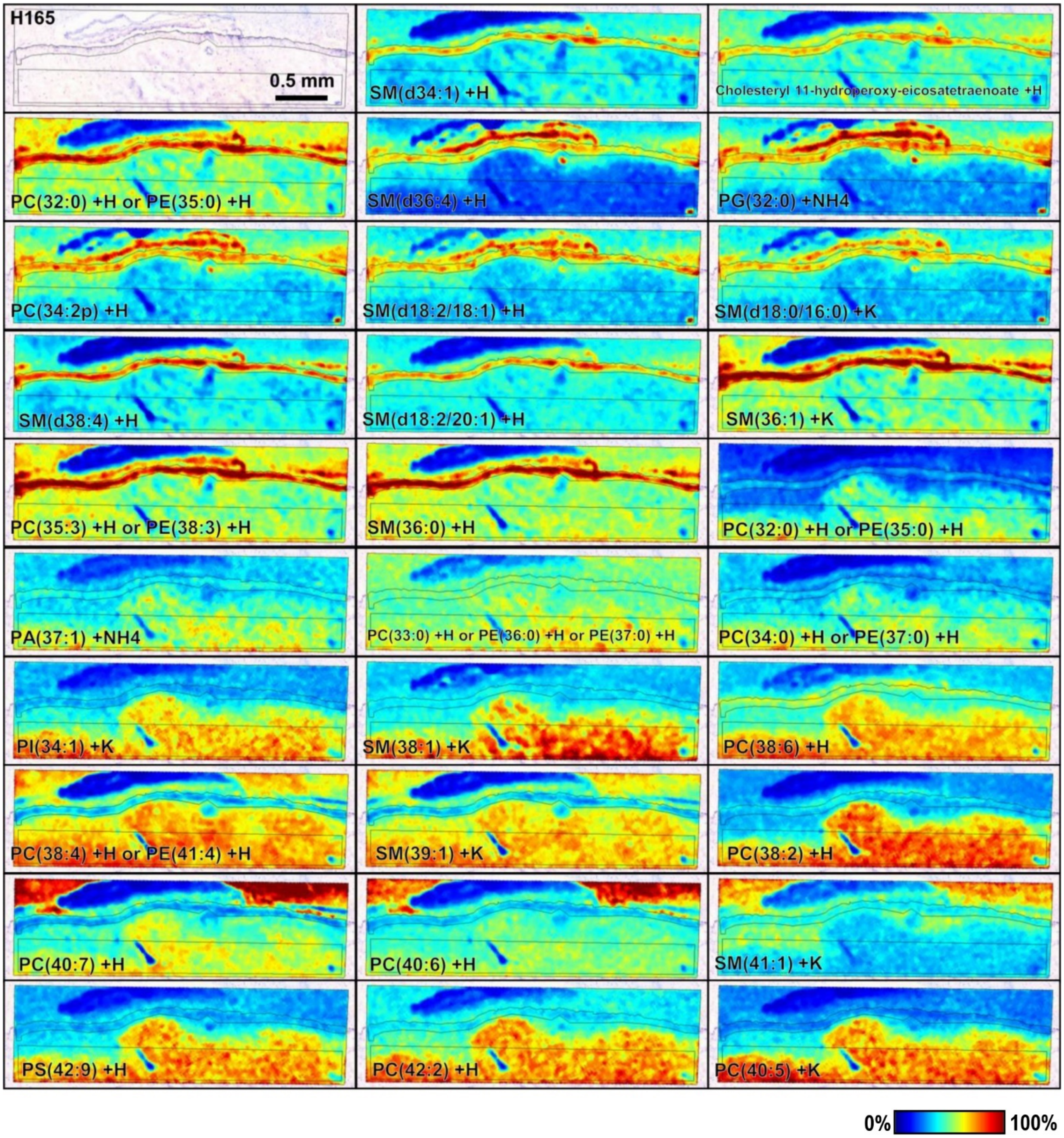

Figure S2. Ion intensity maps for select species that showed differential abundance in the SVZ compared to the CN in positive ion detection mode.

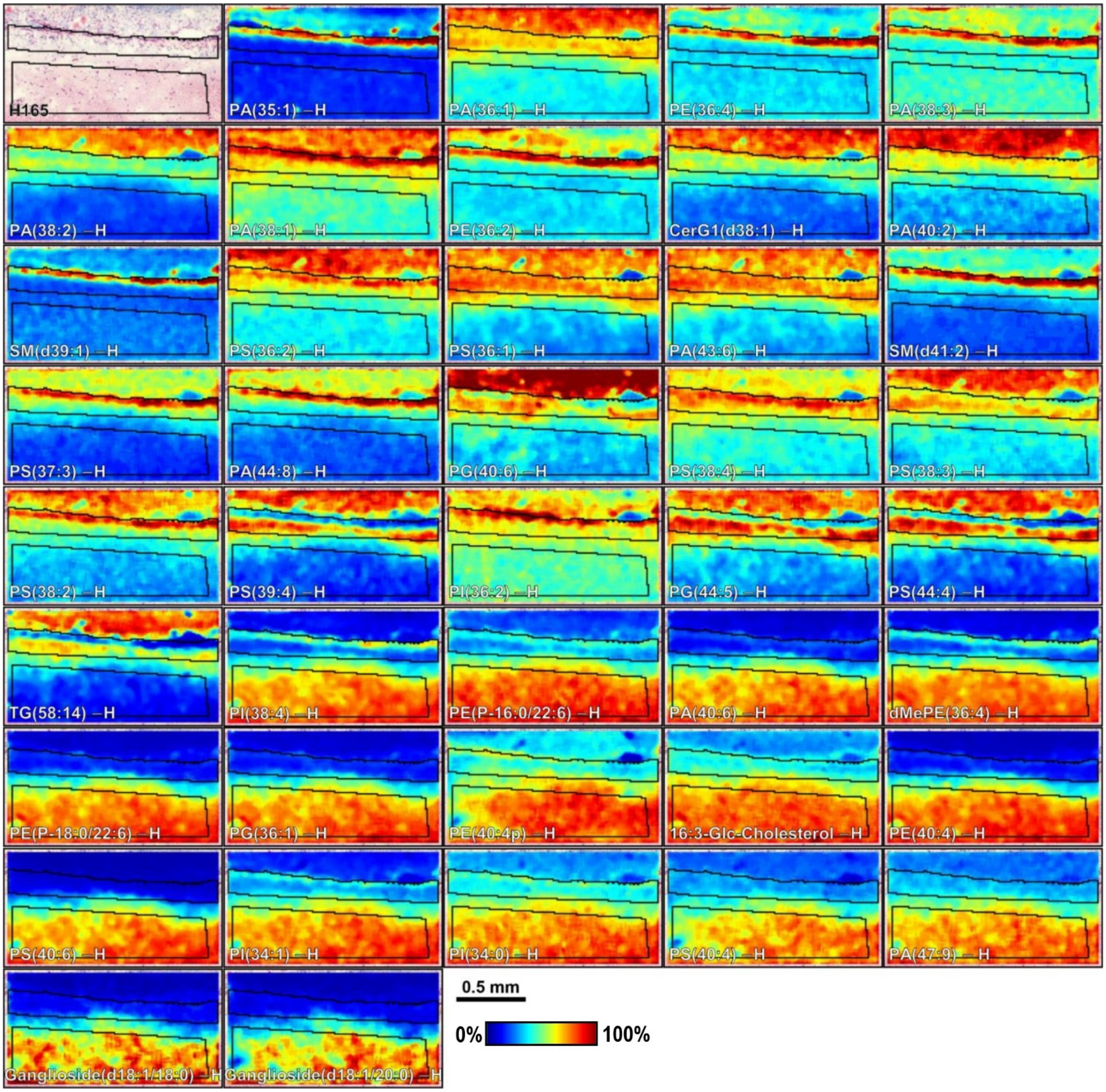

Figure S3. Ion intensity maps for select species that showed differential abundance in the SVZ compared to the CN in negative ion detection mode.

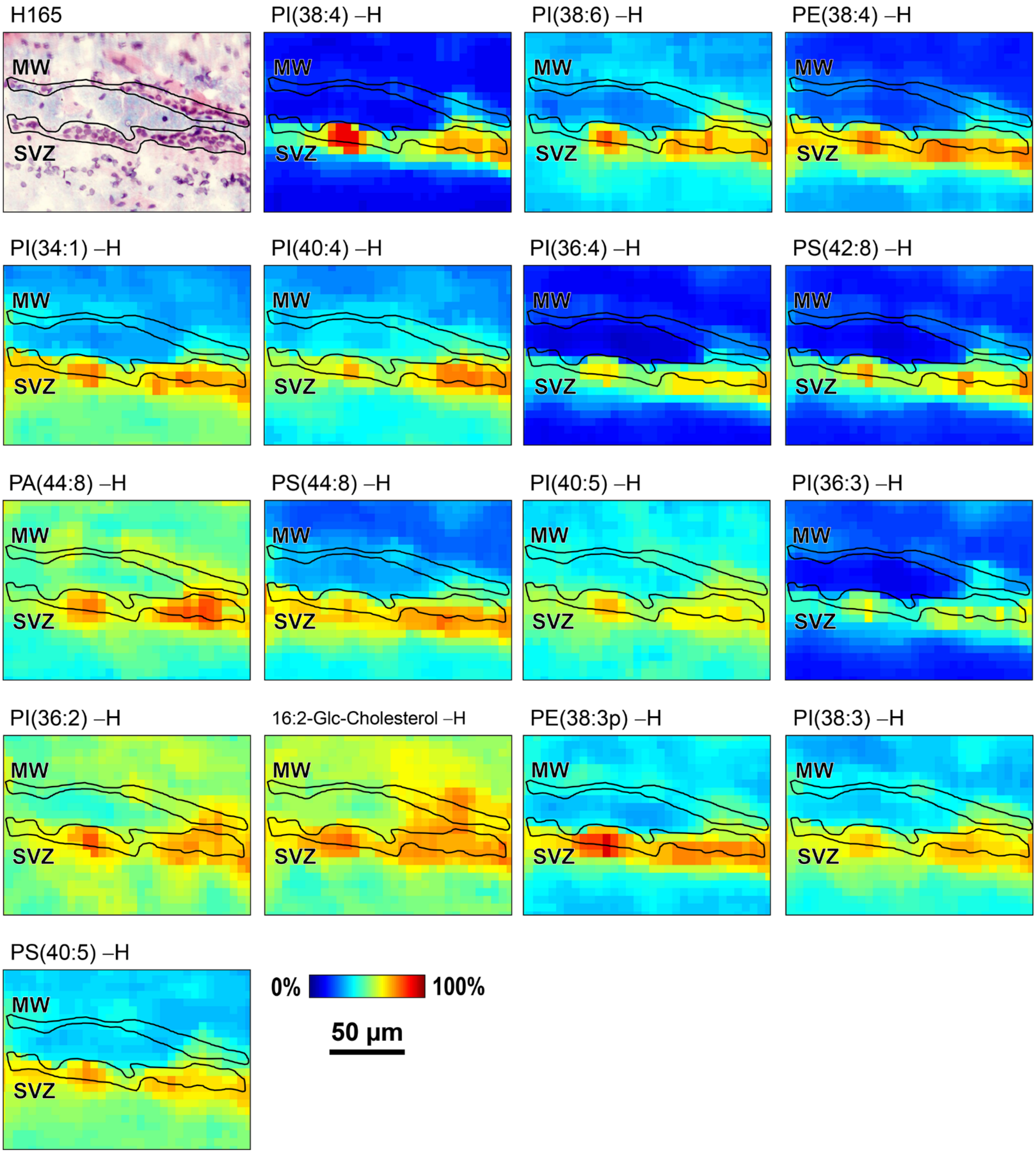

Figure S4. Ion intensity maps for lipids identified to the ependymal layer, comparing intensity in the ependyma of the SVZ (i.e. lateral wall of the ventricle) and the medial wall (MW) of the ventricle in a representative case.

Supplementary Tables

Table S1. Lipid mass signals in positive ionisation mode that show higher abundance in the subventricular zone relative to the caudate nucleus. Co-localisation coefficients (Pearson r), areas under the ROC curve (AUC-ROC) and SVZ/CN signal abundance ratios are expressed as mean ± standard error of the mean for four cases. TOF obs. m/z and FT-ICR obs. m/z refer to the experimentally observed m/z for the corresponding analyte per MALDI-TOF and FT-ICR, respectively. FT-ICR error denotes the difference (in ppm) between the observed accurate mass the predicted m/z of the assignment.

Assignment	TOF obs. m/z	FT-ICR obs. m/z	Predicted m/z	FT-ICR error (ppm)	Co-localisation (r)	AUC-ROC	Abundance (SVZ/CN)
SM(d36:4) +H	725.6	725.561	725.559	3	0.70 ± 0.09	0.93 ± 0.05	3.07 ± 0.52
PG(33:0) +NH4	754.6	754.595	754.597	3	0.68 ± 0.03	0.91 ± 0.02	1.90 ± 0.08
SM(d38:4) +H	753.6	753.592	753.591	1	0.64 ± 0.05	0.87 ± 0.04	1.95 ± 0.11
SM(36:1) +K or PE-Cer(39:1) +K or PE-Cer(382) +K	769.6	769.567	769.562	6	0.64 ± 0.05	0.87 ± 0.04	1.91 ± 0.25
PC(35:3) +H or PE(38:3) +H	770.6	770.57	770.569	1	0.61 ± 0.03	0.86 ± 0.02	1.80 ± 0.19
SM(d44:5) +H	835.7	835.672	835.669	4	0.60 ± 0.06	0.87 ± 0.03	2.05 ± 0.27
PC(34:2p) +H	742.6	742.575	742.575	0	0.59 ± 0.10	0.87 ± 0.06	2.29 ± 0.24
PG(32:0) +NH4	726.6	726.564	726.564	0	0.58 ± 0.13	0.86 ± 0.06	2.66 ± 0.44
SM(d34:1) +H	703.6	703.578	703.575	4	0.56 ± 0.16	0.84 ± 0.11	2.36 ± 0.51
SM(36:0) +H	771.6	771.574	771.578	5	0.50 ± 0.06	0.80 ± 0.04	1.54 ± 0.07
PC(32:0) +H or PE(35:0) +H	720.8	720.594	720.59	6	0.47 ± 0.06	0.78 ± 0.05	1.68 ± 0.11
SM(d18:0/16:0) +K	743.6	743.541	743.546	7	0.45 ± 0.16	0.78 ± 0.10	2.17 ± 0.41
Cholesteryl 11-hydroperoxy-eicosatetraenoate +H	705.8	705.584	705.582	3	0.44 ± 0.04	0.78 ± 0.03	1.59 ± 0.04
SM(d18:2/18:1) +H	727.6	727.576	727.575	1	0.43 ± 0.16	0.77 ± 0.10	2.42 ± 0.44
PC(32:1) +H or PE-NMe(34:1) +H or PE(35:1) +H	732.6	732.558	732.554	5	0.40 ± 0.14	0.74 ± 0.08	1.42 ± 0.16
PE(36:4) +H	740.5	740.524	740.522	3	0.36 ± 0.06	0.73 ± 0.04	1.68 ± 0.08
SM(d38:5) +H	751.6	751.576	751.575	1	0.36 ± 0.04	0.73 ± 0.02	1.47 ± 0.05
PC(36:4e) +H or PE(39:3p) +H	768.6	768.593	768.59	4	0.30 ± 0.04	0.69 ± 0.02	1.24 ± 0.03

Table S2. Lipid mass signals in negative ionisation mode that show higher abundance in the subventricular zone relative to the caudate nucleus. Co-localisation coefficients (Pearson r), areas under the ROC curve (AUC-ROC) and SVZ/CN signal abundance ratios are expressed as mean ± standard error of the mean for four cases. TOF obs. m/z and FT-ICR obs. m/z refer to the experimentally observed m/z for the corresponding analyte per MALDI-TOF and FT-ICR, respectively. FT-ICR error denotes the difference (in ppm) between the observed accurate mass the predicted m/z of the assignment.

Assignment	TOF obs. m/z	FT-ICR obs. m/z	Predicted m/z	FT-ICR error (ppm)	Co-localisation (r)	AUC-ROC	Abundance (SVZ/CN)
PE(36:2p) -H or dMePE(34:2p) -H	726.5	726.547	726.544	4	0.90 ± 0.01	1.00 ± 0.00	4.81 ± 0.78
PA(38:2) -H	727.6	727.531	727.528	4	0.87 ± 0.01	0.99 ± 0.00	2.99 ± 0.41
PA(43:6) -H	789.5	789.551	789.544	9	0.86 ± 0.01	0.99 ± 0.00	2.80 ± 0.35
PS(36:1) -H	788.7	788.548	788.545	4	0.83 ± 0.04	0.98 ± 0.01	3.14 ± 0.49
PE(34:1p) -H or dMePE(32:1p) -H	700.5	700.531	700.529	3	0.82 ± 0.02	0.99 ± 0.00	2.05 ± 0.16
PA(36:1) -H	701.6	701.515	701.513	3	0.82 ± 0.02	0.98 ± 0.01	2.07 ± 0.18
dMePE(34:1p) -H or PE(36:1p) -H	728.7	728.563	728.56	4	0.81 ± 0.03	0.98 ± 0.01	2.41 ± 0.27
CerG1(d38:1) -H	754.7	754.616	754.62	5	0.81 ± 0.03	0.98 ± 0.01	2.70 ± 0.32
PS(37:3) -H	798.8	798.532	798.529	4	0.78 ± 0.02	0.98 ± 0.01	3.90 ± 0.47
PS(38:4) -H	810.6	810.533	810.529	5	0.74 ± 0.02	0.95 ± 0.01	1.85 ± 0.09
PS(38:3) -H	812.8	812.549	812.545	5	0.72 ± 0.03	0.92 ± 0.02	1.73 ± 0.03
SM(d41:2) -H	797.7	797.658	797.654	5	0.71 ± 0.02	0.99 ± 0.01	4.29 ± 0.95
PA(40:2) -H	755.6	755.563	755.56	4	0.71 ± 0.01	0.93 ± 0.01	1.94 ± 0.07
PS(38:2) -H	814.7	814.564	814.56	5	0.70 ± 0.03	0.93 ± 0.02	2.20 ± 0.08
PS(44:4) -H	894.7	894.629	894.623	7	0.69 ± 0.05	0.87 ± 0.04	2.37 ± 0.13
PA(44:8) -H	799.6	799.535	799.528	9	0.68 ± 0.03	0.97 ± 0.01	3.24 ± 0.27
PA(38:1) -H	729.6	729.547	729.544	4	0.68 ± 0.01	0.92 ± 0.01	1.70 ± 0.05
PS(36:2) -H	786.5	786.532	786.529	4	0.66 ± 0.09	0.89 ± 0.06	1.95 ± 0.27
PI(36:2) -H	861.6	861.554	861.55	5	0.66 ± 0.04	0.93 ± 0.03	1.89 ± 0.20
PG(44:5) -H	879.6	879.612	879.612	0	0.65 ± 0.06	0.86 ± 0.04	1.84 ± 0.12
PG(40:6) -H	807.6	807.553	807.555	2	0.64 ± 0.03	0.89 ± 0.02	1.67 ± 0.08
TG(58:14) -H	917.7	917.663	917.667	4	0.63 ± 0.02	0.83 ± 0.02	2.35 ± 0.07
PE(36:2) -H	742.7	742.543	742.539	5	0.63 ± 0.01	0.88 ± 0.02	1.97 ± 0.16
PS(39:4) -H	824.8	824.548	824.545	4	0.61 ± 0.06	0.84 ± 0.04	2.59 ± 0.10
PE(36:4) -H	724.6	724.531	724.529	3	0.55 ± 0.03	0.81 ± 0.03	1.56 ± 0.03
PA(35:1) -H	687.6	687.5	687.497	4	0.54 ± 0.01	0.83 ± 0.03	2.63 ± 0.14
PA(38:3) -H	725.7	725.516	725.513	4	0.44 ± 0.06	0.73 ± 0.04	1.38 ± 0.01
SM(d39:1) -H	771.7	771.642	771.639	4	0.42 ± 0.02	0.71 ± 0.04	2.34 ± 0.43

Table S3. Lipid mass signals in positive ionisation mode that show lower abundance in the subventricular zone relative to the caudate nucleus. Co-localisation coefficients (Pearson r), areas under the ROC curve (AUC-ROC) and SVZ/CN signal abundance ratios are expressed as mean ± standard error of the mean for four cases. TOF obs. m/z and FT-ICR obs. m/z refer to the experimentally observed m/z for the corresponding analyte per MALDI-TOF and FT-ICR, respectively. FT-ICR error denotes the difference (in ppm) between the observed accurate mass the predicted m/z of the assignment.

Assignment	TOF obs. m/z	FT-ICR obs. m/z	Predicted m/z	FT-ICR error (ppm)	Co-localisation (r)	AUC-ROC	Abundance (SVZ/CN)
PC(32:0) +H or PE(35:0) +H	734.6	734.573	734.569	5	-0.66 ± 0.08	0.07 ± 0.02	0.48 ± 0.02
PC(40:6) +H	834.6	834.606	834.601	6	-0.58 ± 0.07	0.11 ± 0.24	0.45 ± 0.04
PC(34:0) +H or PE(37:0) +H	762.6	762.607	762.601	8	-0.55 ± 0.06	0.13 ± 0.02	0.61 ± 0.03
PC(38:4) +H or PE(41:4) +H	810.7	810.604	810.601	4	-0.52 ± 0.09	0.17 ± 0.05	0.64 ± 0.07
PA(37:1) +NH4	736.6	736.58	736.585	7	-0.51 ± 0.08	0.15 ± 0.04	0.52 ± 0.03
PC(38:2) +H	814.6	814.638	814.632	7	-0.50 ± 0.04	0.11 ± 0.21	0.48 ± 0.06
PC(38:6) +H	806.6	806.574	806.569	6	-0.49 ± 0.08	0.17 ± 0.03	0.60 ± 0.05
PC(40:5) +K	874.6	874.579	874.572	8	-0.48 ± 0.11	0.11 ± 0.28	0.43 ± 0.11
SM(39:1) +K	811.7	811.607	811.609	2	-0.48 ± 0.08	0.19 ± 0.05	0.64 ± 0.07
PS(42:9) +H	858.6	858.531	858.528	3	-0.42 ± 0.07	0.11 ± 0.26	0.51 ± 0.10
PC(40:7) +H	832.6	832.588	832.585	4	-0.41 ± 0.13	0.11 ± 0.23	0.62 ± 0.11
SM(38:1) +K	797.6	797.598	797.593	6	-0.39 ± 0.16	0.24 ± 0.12	0.67 ± 0.18
PI(34:1) +K	875.6	875.508	875.505	3	-0.35 ± 0.09	0.11 ± 0.29	0.54 ± 0.15
PC(39:2p) +H	812.7	-	812.653	2	-0.30 ± 0.02	0.11 ± 0.20	0.76 ± 0.01
PC(33:0) +H or PE(36:0) +H or PE(37:0) +H	748.6	748.589	748.585	5	-0.28 ± 0.03	0.31 ± 0.02	0.71 ± 0.03
PC(42:2) +H	870.6	870.7	870.695	6	-0.22 ± 0.04	0.11 ± 0.27	0.61 ± 0.04
SM(41:1) +K	839.7	839.641	839.64	1	-0.15 ± 0.02	0.11 ± 0.25	0.79 ± 0.02

Table S4. Lipid mass signals in negative ionisation mode that show lower abundance in the subventricular zone relative to the caudate nucleus. Co-localisation coefficients (Pearson r), areas under the ROC curve (AUC-ROC) and SVZ/CN signal abundance ratios are expressed as mean ± standard error of the mean for four cases. TOF obs. m/z and FT-ICR obs. m/z refer to the experimentally observed m/z for the corresponding analyte per MALDI-TOF and FT-ICR, respectively. FT-ICR error denotes the difference (in ppm) between the observed accurate mass the predicted m/z of the assignment.

Assignment	TOF obs. m/z	FT-ICR obs. m/z	Predicted m/z	FT-ICR error (ppm)	Co-localisation (r)	AUC-ROC	Abundance (SVZ/CN)
PC(37:6) -H or PE(40:6) -H	790.6	790.542	790.539	4	-0.90 ± 0.01	0.00 ± 0.00	0.23 ± 0.04
PE(P-18:0/22:6) -H	774.7	774.548	774.544	5	-0.89 ± 0.01	0.01 ± 0.00	0.30 ± 0.04
PE(40:4) -H	794.7	794.574	794.571	4	-0.89 ± 0.01	0.01 ± 0.00	0.29 ± 0.04
PA(40:6) -H	747.6	747.5	747.497	4	-0.87 ± 0.01	0.01 ± 0.00	0.33 ± 0.05
PS(40:6) -H	834.7	834.532	834.529	4	-0.86 ± 0.03	0.01 ± 0.01	0.21 ± 0.04
PG(36:1) -H	775.6	775.551	775.55	1	-0.82 ± 0.07	0.04 ± 0.03	0.33 ± 0.05
PE(40:5) -H or dMePE(38:5) -H	792.6	792.547	792.555	10	-0.82 ± 0.06	0.03 ± 0.02	0.34 ± 0.03
PE(P-16:0/22:6) -H	746.5	746.516	746.513	4	-0.80 ± 0.02	0.03 ± 0.01	0.53 ± 0.04
PS(40:4) -H	838.8	838.565	838.56	6	-0.79 ± 0.01	0.03 ± 0.01	0.46 ± 0.04
GM1(d20:1-C/18:0) -H	1573	1572.913	1572.901	8	-0.76 ± 0.05	0.03 ± 0.01	0.26 ± 0.03
PE(38:3) -H	768.7	768.547	768.555	10	-0.76 ± 0.04	0.05 ± 0.02	0.54 ± 0.03
PI(38:4) -H	885.6	885.554	885.55	5	-0.75 ± 0.04	0.07 ± 0.02	0.51 ± 0.06
PI(18:0) -H or 6-O-(1-O-stearoyl-sn-glycero-3-phosphono)-1D-myo-inositol -H	599.5	599.322	599.32	3	-0.75 ± 0.02	0.05 ± 0.01	0.54 ± 0.06
16:3-Glc-Cholesterol	779.7	779.582	779.583	1	-0.75 ± 0.02	0.06 ± 0.01	0.60 ± 0.04
dMePE(38:3) -H or PE(40:3)-H	796.6	796.579	796.586	9	-0.75 ± 0.02	0.05 ± 0.01	0.56 ± 0.04
Fucalpha1-2Galbeta1-3(Fucalpha1-4)GlcNAcbeta1-3Galbeta1-4Glcbeta-Cer(d18:1/18:0) -H or Fucalpha1-2Galbeta1-4(Fucalpha1-3)GlcNAcbeta1-3Galbeta1-4Glcbeta-Cer(d18:1/18:0) -H	1545.9	1545.883	1545.89	5	-0.73 ± 0.05	0.05 ± 0.02	0.31 ± 0.04
PA(47:9) -H	839.6	839.567	839.56	8	-0.73 ± 0.04	0.05 ± 0.02	0.49 ± 0.04
PE(40:4p) -H	778.8	778.579	778.576	4	-0.73 ± 0.01	0.06 ± 0.01	0.61 ± 0.02
dMePE(36:4) -H	766.5	766.542	766.539	4	-0.72 ± 0.09	0.09 ± 0.05	0.49 ± 0.03
PI(34:1) -H	835.6	835.536	835.534	2	-0.72 ± 0.07	0.07 ± 0.03	0.55 ± 0.03
GM1(d18:1-C/18:0) -H	1544.8	1544.88	1544.869	7	-0.72 ± 0.06	0.06 ± 0.06	0.31 ± 0.04
PC(35:1) -H or PE(38:1) -H	772.6	772.589	772.586	4	-0.72 ± 0.03	0.07 ± 0.02	0.63 ± 0.02
Fucalpha1-2Galbeta1-3(Fucalpha1-4)GlcNAcbeta1-3Galbeta1-4Glcbeta-Cer(d18:1/20:0) -H or Fucalpha1-2Galbeta1-4(Fucalpha1-3)GlcNAcbeta1-3Galbeta1-4Glcbeta-Cer(d18:1/20:0) -H	1573.9	1573.915	1573.921	4	-0.69 ± 0.11	0.08 ± 0.06	0.30 ± 0.07
PS(40:5) -H	836.6	836.537	836.545	10	-0.69 ± 0.06	0.08 ± 0.02	0.61 ± 0.03
PI(34:0) -H	837.7	837.555	837.55	6	-0.68 ± 0.04	0.09 ± 0.02	0.61 ± 0.04

Table S5. Lipid mass signals co-localised with the ependymal layer. Co-localisation coefficients (Pearson r) are expressed as mean ± standard error of the mean for four cases. TOF obs. m/z and FT-ICR obs. m/z refer to the experimentally observed m/z for the corresponding analyte per MALDI-TOF and FT-ICR, respectively. FT-ICR error denotes the difference (in ppm) between the observed accurate mass the predicted m/z of the assignment.

Assignment	TOF obs. m/z	FT-ICR obs. m/z	Predicted m/z	FT-ICR error (ppm)	Co-localisation (r)
PI(38:4) -H	871.7	871.574	871.571	3	0.74 ± 0.05
PI(38:6) -H	881.6	881.522	881.519	3	0.71 ± 0.03
PE(38:4) -H	766.5	766.542	766.539	4	0.58 ± 0.08
PI(34:1) -H	835.6	835.536	835.534	2	0.57 ± 0.11
PI(40:4) -H	913.6	913.586	913.581	5	0.57 ± 0.05
PI(36:4) -H	857.7	857.522	857.519	3	0.56 ± 0.04
PS(42:8) -H	858.6	858.526	858.529	3	0.56 ± 0.04
PA(44:8) -H	799.6	799.535	799.528	9	0.55 ± 0.07
PS(44:8) -H	886.5	886.557	886.56	3	0.55 ± 0.06
PI(40:5) -H	911.7	911.571	911.566	5	0.55 ± 0.06
PI(36:3) -H	859.6	859.542	859.534	9	0.54 ± 0.06
PI(36:2) -H	861.6	861.554	861.55	5	0.54 ± 0.06
16:2-Glc-Cholesterol -H	781.6	781.599	781.599	0	0.54 ± 0.04
PE(38:3p) -H	752.6	752.564	752.56	5	0.53 ± 0.06
PI(38:3) -H	887.6	887.559	887.566	8	0.53 ± 0.04
PI-Cer(34:0) +Na	820.6	820.53	820.531	1	0.50 ± 0.06
PC(38:7) +H	804.6	804.556	804.554	2	0.49 ± 0.07
PS(40:5) -H	836.6	836.551	836.545	7	0.48 ± 0.18
PC(40:7) +H	832.6	832.588	832.585	4	0.48 ± 0.07
PA(O-20:0/22:4) +K	805.6	805.559	805.551	10	0.42 ± 0.07
PG(40:7) +H	821.6	821.534	821.533	1	0.39 ± 0.07
PE(24:4/17:2) +H or PC(38:6) +H	806.6	806.574	806.569	6	0.35 ± 0.09

Table S6. Lipid mass signals co-localised with the hypocellular layer. Co-localisation coefficients (Pearson r) are expressed as mean ± standard error of the mean for four cases. TOF obs. m/z and FT-ICR obs. m/z refer to the experimentally observed m/z for the corresponding analyte per MALDI-TOF and FT-ICR, respectively. FT-ICR error denotes the difference (in ppm) between the observed accurate mass the predicted m/z of the assignment.

Assignment	TOF obs. m/z	FT-ICR obs. m/z	Predicted m/z	FT-ICR error (ppm)	Co-localisation (r)
PE(36:4) -H	738.6	738.51	738.508	3	0.59 ± 0.04
PE(38:5) -H	764.6	764.527	764.524	4	0.59 ± 0.03
SM(35:1) -H or PE-Cer(38:1) -H	715.6	715.579	715.576	4	0.58 ± 0.06
PE(34:1) -H	716.7	716.526	716.524	3	0.58 ± 0.06
GlcCer(32:1) -H	670.7	670.52	670.526	9	0.57 ± 0.11
dMePE(32:0) -H	718.6	718.542	718.539	4	0.57 ± 0.04
PI(38:5) -H	883.7	883.539	883.534	6	0.57 ± 0.04
PE(36:2) -H	742.7	742.543	742.539	5	0.56 ± 0.05
PA(35:1) -H	687.6	687.499	687.497	3	0.56 ± 0.03
DG(22:5/22:6/0:0) -H	713.6	713.515	713.515	0	0.56 ± 0.03
PA(34:1) -H	673.7	673.483	673.481	3	0.53 ± 0.06
PE(38:5p) -H	748.7	748.532	748.529	4	0.53 ± 0.02
CerP(d18:1/18:0) -H	644.6	644.504	644.503	2	0.52 ± 0.08
PE(38:4p) -H	750.6	750.547	750.544	4	0.51 ± 0.07
PE(34:2) -H	714.7	714.51	714.508	3	0.51 ± 0.03
PC(33:1) -H or PE(36:1) -H	744.6	744.558	744.555	4	0.51 ± 0.02
GlcCer(30:1) -H	642.5	642.489	642.495	9	0.50 ± 0.12
SM(d38:4) +H	753.6	753.591	753.591	0	0.45 ± 0.07
SM(d36:1) +K or PE-Cer(d39:1) +K	769.6	769.567	769.562	6	0.45 ± 0.07
PC(32:0e) +H	720.8	720.594	720.59	6	0.41 ± 0.04
SM(d34:0) +H	705.8	705.584	705.591	10	0.39 ± 0.06
SM(d36:1) +H	731.6	731.61	731.606	5	0.38 ± 0.13

Table S7. Lipid mass signals co-localised with the myelin layer. Co-localisation coefficients (Pearson r) are expressed as mean ± standard error of the mean for four cases. TOF obs. m/z and FT-ICR obs. m/z refer to the experimentally observed m/z for the corresponding analyte per MALDI-TOF and FT-ICR, respectively. FT-ICR error denotes the difference (in ppm) between the observed accurate mass the predicted m/z of the assignment.

Assignment	TOF obs. m/z	FT-ICR obs. m/z	Predicted m/z	FT-ICR error (ppm)	Co-localisation (r)
Sulfatide (3'-sulfo)Galbeta-Cer(d18:1/24:1(15Z)(2OH)) -H	904.8	904.624	904.619	6	0.76 ± 0.03
Sulfatide (3'-sulfo)Galbeta-Cer(d18:1/24:0(2OH)) -H	906.7	906.639	906.635	4	0.74 ± 0.04
TG(58:1) -H	917.7	917.662	917.667	5	0.73 ± 0.06
Sulfatide (3'-sulfo)Galbeta-Cer(d18:1/24:1(15Z)) -H	888.7	888.628	888.624	5	0.73 ± 0.04
Sulfatide (3'-sulfo)Galbeta-Cer(d18:1/24:0) -H	890.8	890.645	890.64	6	0.73 ± 0.04
PS(39:4) -H	824.8	824.548	824.545	4	0.73 ± 0.01
Sulfatide (3'-sulfo)Galbeta-Cer(d18:1/22:0(2OH)) -H	878.8	878.608	878.603	6	0.70 ± 0.06
TG(18:4/18:4/20:5) -H	891.7	891.647	891.651	4	0.69 ± 0.08
PS(44:4) -H	894.7	894.629	894.623	7	0.68 ± 0.07
PG(44:5) -H	879.6	879.612	879.612	0	0.63 ± 0.09
PC(36:1) +H	788.7	788.621	788.616	6	0.45 ± 0.15
CL(78:0) +H	1550.2	1550.215	1550.199	10	0.42 ± 0.17
PG(O-20:0/22:0) +H	849.7	849.688	849.694	7	0.39 ± 0.22
TG(50:4) +K	865.7	865.662	865.668	7	0.34 ± 0.14
PC(32:1) +H	732.6	732.557	732.554	4	0.33 ± 0.16
TG(18:4/18:4/18:4) +H	867.7	867.656	867.65	7	0.33 ± 0.11
